# Severe ceftazidime-induced drug reaction with eosinophilia and systemic symptoms (DRESS)

**DOI:** 10.1186/1710-1492-7-S2-A37

**Published:** 2011-11-14

**Authors:** Matthieu Picard, Philippe Bégin, Jean Paradis, Anne Des Roches, Louis Paradis, Françoise Le Deist

**Affiliations:** 1 Department of medicine, Centre Hospitalier de l’Université de Montréal (CHUM), Montreal, Canada, H2L 4M1; 2 Department of pediatrics, CHU Sainte-Justine, Université de Montréal, Montreal, Canada, H3T 1C5; 3 Department of microbiology and immunology, CHU Sainte-Justine, Université de Montréal, Montreal, Canada, H3T 1C5

## Background

Drug reaction with eosinophilia and systemic symptoms (DRESS) is among the most severe forms of drug hypersensitivity [1]. Antiepileptics are by far the most commonly reported causative drugs [2]. Antibiotics have seldom been reported apart from minocycline [3].

## Case

We describe a 55 years old female who developed DRESS with acute liver and kidney failure after being treated with ceftazidime and vancomycine. She was successfully treated with corticosteroids although while tapering prednisone she experienced a recurrence of the skin eruption without any systemic symptoms. She was taken off corticosteroids after 9 months of treatment.

## In vitro tests

Fourteen months after the drug reaction, in vitro tests to identify the causal agent were performed. The lymphocyte transformation test (LTT) showed a marked proliferation to ceftazidime (stimulation index (SI): 17 at 100mcg/mL). CD25 was upregulated on CD4+ (induced expression: 17%) and CD8+ (induced expression: 8%) T cells as shown by flow cytometry when cultured with ceftazidime 50 mcg/mL. IFN-γ was markedly elevated in the supernatant of peripheral blood mononuclear cells (PBMC) cultured with ceftazidime 50 mcg/mL when compared to the control media (946 vs 13 pg/mL). Vancomycin did not induce a significant response when compared to the control media in the flow cytometry and the IFN-γ assays.

## Conclusions

This is the first report of ceftazidime-induced DRESS to be subsequently proven by allergy tests. This case illustrates the importance of considering every susceptible drug as the potential etiologic agent. We also show the usefulness of in vitro tests in their identification.
